# Different serological cross-reactivity of *Trypanosoma rangeli *forms in *Trypanosoma cruzi*-infected patients sera

**DOI:** 10.1186/1756-3305-1-20

**Published:** 2008-07-08

**Authors:** Milene H de Moraes, Alessandra A Guarneri, Fabiana P Girardi, Juliana B Rodrigues, Iriane Eger, Kevin M Tyler, Mário Steindel, Edmundo C Grisard

**Affiliations:** 1 Departamento de Microbiologia e Parasitologia, Universidade Federal de Santa Catarina, 88040-900, Florianópolis, Santa Catarina, Brazil; 2 Instituto René Rachou, Fiocruz, Belo Horizonte, Minas Gerais, Brazil; 3 Universidade do Vale do Itajaí, Itajaí, Santa Catarina, Brazil; 4 Biomedical Research Centre, School of Medicine, Health Policy and Practice, University of East Anglia, Norwich, Norfolk, UK

## Abstract

**Background:**

American Trypanosomiasis or Chagas disease is caused by *Trypanosoma cruzi *which currently infects approximately 16 million people in the Americas causing high morbidity and mortality. Diagnosis of American trypanosomiasis relies on serology, primarily using indirect immunofluorescence assay (IFA) with *T. cruzi *epimastigote forms. The closely related but nonpathogenic *Trypanosoma rangeli *has a sympatric distribution with *T. cruzi *and is carried by the same vectors. As a result false positives are frequently generated. This confounding factor leads to increased diagnostic test costs and where false positives are not caught, endangers human health due to the toxicity of the drugs used to treat Chagas disease.

**Results:**

In the present study, serologic cross-reactivity between the two species was compared for the currently used epimastigote form and the more pathologically relevant trypomastigote form, using IFA and immunoblotting (IB) assays. Our results reveal an important decrease in cross reactivity when *T. rangeli *culture-derived trypomastigotes are used in IFA based diagnosis of Chagas disease. Western blot results using sera from both acute and chronic chagasic patients presenting with cardiac, indeterminate or digestive disease revealed similar, but not identical, antigenic profiles.

**Conclusion:**

This is the first study addressing the serological cross-reactivity between distinct forms and strains of *T. rangeli *and *T. cruzi *using sera from distinct phases of the Chagasic infection. Several *T. rangeli*-specific proteins were detected, which may have potential as diagnostic tools.

## Background

*Trypanosoma rangeli *and *Trypanosoma cruzi *are closely related and sympatric protozoan parasites that infect triatomine bugs, humans and a variety of sylvatic and domestic mammals in overlapping regions of both South and Central America [1-3]. *T. rangeli *is nonpathogenic to humans while *T. cruzi *is the etiological agent of Chagas disease and infects over 16 million people in the new world [4]. Unsurprisingly, given the sympatric distribution and shared vector and host range, mixed infections are observed [1-3].

*T. rangeli *is considered a parasite of biological and epidemiological interest due to the serological cross-reactivity with *T. cruzi*, which has been the subject of great controversy [5-8]. Several reports pointed out the sharing of antigenic epitopes by *T. cruzi *and *T. rangeli *[9-12]. However, these studies were performed with sera from animals immunized with *T. rangeli *epimastigotes rather than infected or immunized with the trypomastigote forms present in infected hosts [9-12]. It is widely accepted to have an outstanding but underestimated impact on the diagnosis of Chagas disease [2,9-14].

A recent study on *T. cruzi *serodiagnosis reported the absence of cross-reaction of *T. rangeli-*infected patient's sera in some commercially available ELISA tests [15]. Despite the fact that PCR-based diagnosis can provide accurate discrimination of *T. cruzi *from *T. rangeli *[2,3], neither method is widespread in the diagnostic laboratories of endemic areas where IFA of cultured *T. cruzi *epimastigote forms with patient sera remains the screening method of choice [16-18]. The use of at least two serological tests based on different methods, mandatory in Brazil for almost two decades now [17], have enhanced the sensitivity detection. Comparative studies of the antigenic composition of culture-derived *T. cruzi *and *T. rangeli *epimastigote forms indicate that these parasites share approximately 60% of their soluble antigenic composition [5,7,11] often resulting in a significant level of inconclusive results [17].

Epimastigote forms are not seen in the blood of infected individuals and are therefore not the form against which antibody develops, rather the forms seen on blood smears are normally the trypomastigote forms. We considered therefore, whether serum screening using trypomastigotes from culture, rather than epimastigotes, would help to reduce misdiagnosis. We extended these studies to examine the expression of cross-reactive proteins between life cycle stages in order to reveal candidate proteins for molecular diagnostics, pathogenicity factors and possible vaccines.

## Results

**Figure 1:**
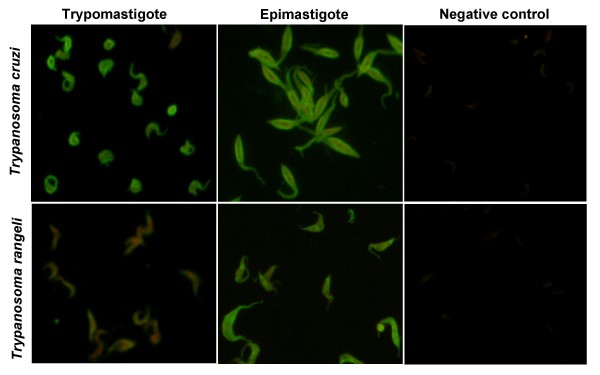
**The use of trypomastigotes give better specificity than epimastigotes by IFA**. Indirect immunofluorescence assay using *Trypanosoma cruzi *and *Trypanosoma rangeli *trypomastigote or epimastigote forms as antigens and serum (1:80) from a chronic chagasic patient with the cardiac form of the disease. Negative controls consist of epimastigotes and non-infected patient serum (1:40).

**Figure 2:**
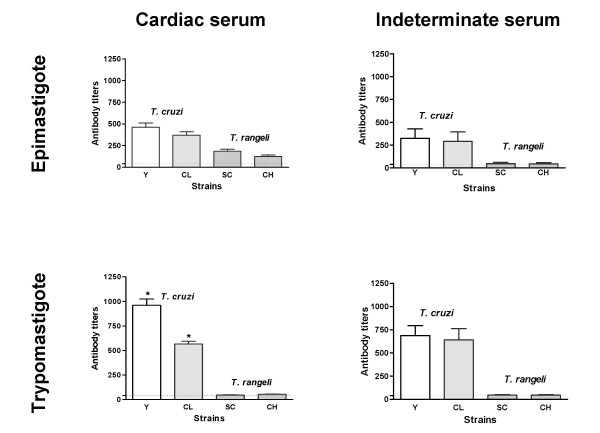
**Trypomastigotes discriminate *Trypanosoma cruzi *from *Trypanosoma rangeli *over an increased titre range**. Comparison of antibody titers obtained for chronic chagasic patients serum with cardiac or indeterminate forms tested against epimastigote or trypomastigote forms of *T. cruzi *Y (open bar) and CL (dotted bar) strains and *T. rangeli *Choachi (vertical lined bar) and SC-58 (horizontal lined bar) strains in indirect immunofluorescence assays. Experiments were performed in duplicates and vertical bars above data indicate mean standard error. Horizontal dotted lines indicate the 1:40 cut-off.

Independent of the clinical form, all chagasic sera tested reacted with both *T. cruzi *and *T. rangeli *epimastigotes in IFA (Figs. 1 and 2). Considering reactions with titers over 1:40 as positive, cross-reactions were observed for 88.46% of the serum from patients with the cardiac form of the Chagas disease (Fig. 2). Interestingly, the use of *T. rangeli *trypomastigote forms as antigen reduced the cross-reactivity of serum from patients with the cardiac form of the Chagas disease to 30.76% (8/26). The titres as well as the fluorescence intensities were variable depending on the parasite species and/or strains used but, irrespective of the form used as antigen, higher titres were obtained for *T. cruzi *strains (Fig. 2).

Except for the distinct and significant reactivity (p < 0.05) of serum from patients with the cardiac form of the Chagas disease with *T. cruzi *trypomastigotes from the CL or Y strains, no other significant intra-specific variation of the reaction titres (p > 0.05) was observed among the strains studied and the fluorescence pattern revealed was homogeneous (Fig. 2). However, inter-specific comparisons revealed a variable fluorescence pattern and significantly different (p < 0.001) reaction titres between *T. cruzi *and *T. rangeli *strains for the tested sera, especially when using trypomastigotes as antigens (Fig. 2).

A representation of the fluorescence patterns observed in IFA is shown in Figure 1. Both *T. cruzi *forms showed a fluorescence pattern varying from homogeneous cellular, including nucleus and kinetoplast when tested against sera from patients with indeterminate form of the disease (data not shown), to strong plasma membrane labeling alone when tested with serum from patients with cardiac involvement (Fig. 1). Sera from all chagasic patients showed strong membrane fluorescence when tested against *T. rangeli *epimastigotes (Fig. 1) although, sera from the indeterminate form patients that reacted with *T. cruzi *epimastigote forms at 1:40 or 1:80 dilutions resulted in negative results when tested against *T. rangeli *epimastigotes at these concentrations (Data not shown).

As expected, chagasic sera gave the strongest reaction with *T. cruzi *trypomastigotes regardless of patient clinical history (Fig. 2). The use of trypomastigote forms from both species in IFA showed a significant decrease (p < 0.001) in cross-reactivity when compared to the results obtained for epimastigote forms (Fig. 2). Also, a significant intra-specific variation (p < 0.05) of the reaction titers was observed among epimastigotes of the distinct *T. cruzi *strains according to the testing sera (cardiac or indeterminate), which was not observed among the trypomastigote forms (Fig. 2).

Sera from chronic chagasic patients (cardiac or indeterminate forms) showing borderline titers of 1:40 or 1:80 against *T. cruzi *epimastigotes, which did not react with *T. rangeli *epimastigotes, revealed negative results in IFA with both *T. rangeli *and *T. cruzi *trypomastigotes.

**Figure 3:**
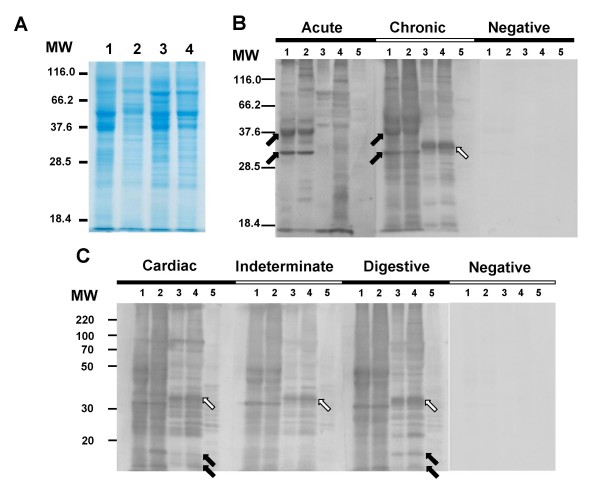
**Immunoblot profiles of *Trypanosoma cruzi *(Y strain) and *Trypanosoma rangeli *(Choachi strain) with chagasic sera**. Total protein extracts resolved in a 12% SDS-PAGE and stained by Comassie brilliant blue (A) and the immunoblot analysis of the same lysates using sera from acute and chronic chagasic patients (B) or using sera from chronic chagasic patients with the cardiac, indeterminate or digestive forms of the disease (C). On panel B, arrows indicates proteins recognized by chagasic sera (acute and/or chronic) on *T. cruzi *(dark arrows) or on *T. rangeli *(white arrow) extracts. Arrows in panel C indicates a *T. rangeli *11 and 15 kDa proteins exclusively recognized by the cardiac and digestive serum in both *T. rangeli *forms (dark arrows) and a 35 kDa protein (white arrows) recognized by all sera from *T. cruzi*-infected patients. Lanes 1 and 2 = *T. cruzi *Y strain epimastigote and trypomastigote forms and lanes 3 and 4 = *T. rangeli *Choachi strain epimastigote and trypomastigote forms, respectively. Lane 5 = Vero cells extract; MW = molecular weight marker (kDa). Asterisks indicates significant differences (p < 0.05).

Comparative SDS-PAGE analysis revealed a protein pattern varying from 24 to 110 kDa for both *Trypanosoma *species (Fig. 3A). Epimastigote forms gave similar patterns, sharing a large number of prominent and faint bands. For trypomastigotes though, it was possible to visualize differences in the electrophoretic profiles (Fig. 3A, lanes 2 and 4). No major differences were observed among the profiles of both *T. rangeli *forms, however, the profile of *T. cruzi *epimastigotes revealed two major proteins around 36 and 52 kDa, as also observed on both *T. rangeli *forms, which were present in much lower intensity on the *T. cruzi *trypomastigotes profile (Fig. 3A).

Immunoblot analysis using all acute and chronic chagasic patient sera recognized a wide range of proteins in both parasite species and strains (data not shown), revealing a quite distinct inter-specific pattern. A representation of the obtained results is shown on Fig. 3B. Sera from patients in the acute phase of Chagas disease recognized fewer proteins on *T. rangeli *extracts than sera from chronic chagasic patients. Two proteins of approximately 32 and 40 kDa were strongly recognized by chagasic sera (acute and chronic) on *T. cruzi *extracts (Fig. 3B, dark arrows), but were not detected in *T. rangeli *lysates by acute sera. A 32 kDa protein was, though, weakly recognized in chronic patient sera. Also, some intra-specific differences on antigenic profile between *T. cruzi *Y strain epimastigote and trypomastigote forms were observed (Fig. 3B).

Signal intensity for several *T. rangeli *antigens was variable between chagasic sera. Interestingly, though, a very strong band of approximately 35 kDa was recognized in both *T. rangeli *forms by sera from chronic patients (Fig. 3B, white arrow).

We further considered whether chagasic sera from different pathologies showed similar antigenic profiles by utilizing sera from patients presenting the cardiac, indeterminate or digestive forms of the disease (Fig. 3C). Our results revealed very similar profiles for *T. cruzi *epimastigotes and trypomastigotes but a quite distinct protein patterns for *T. rangeli *epimastigotes and trypomastigotes, independently of the tested sera. Importantly, a 35 kDa protein was recognized strongly by all chagasic sera (Fig. 3C, white arrows). Two proteins of around 11 and 15 kDa were recognized by the cardiac and digestive serum in both *T. rangeli *forms, but not by the indeterminate sera (Fig. 3C, black arrows).

## Discussion

Despite the proven cross-reactions and the existence of more sensitive and reliable techniques for diagnosis such as ELISA associated to the use of recombinant antigens, IFA is still the most widely used method for Chagas disease diagnosis in Brazil, as in several Latin American Countries [16-18]. Thus, in the present study the serological cross-reactivity between *T. cruzi *and *T. rangeli*, epimastigote and trypomastigote forms, was evaluated by IFA and IB assays using sera of both acute and chronic chagasic patients with different clinical manifestations.

Former studies have shown the serological cross-reactivity between *T. cruzi *and *T. rangeli *epimastigotes by IFA, ELISA and/or IB assays, pointing that such cross-reactivity is due the great number of soluble antigens shared by both parasites [5,7,9-12,19]. Furthermore, sera from *T. cruzi*-infected patients can cross-react with other trypanosomatid species such as *T. evansi *[20] and *Leishmania *spp. [15] as demonstrated by western-blot, ELISA and TESA-Blot, including some commercially available kits.

In the present study we have confirmed cross-reactivity in a range of distinct serum samples and also observed that *T. cruzi *and *T. rangeli *epimastigotes reacted with all tested sera regardless of the clinical presentation of the patient. However, the fluorescence intensity and pattern produced by the sera in IFA on parasites cells appeared to be variable depending on the parasite species and life-cycle stage and upon the type of serum used. Such differences may be attributed both to the distinct antibody profiles of the sera, which in turn can be related to the disease form and/or to the patient immune response, as well as to the differences in gene expression and antigenic composition of the parasites themselves.

It is well established that distinct profiles of anti-*T. cruzi *antibodies may be elicited by patients with different clinical presentations of the disease [21-23]. This variable response pattern can be due to a variety of immune response related mechanisms, such as the regulation of immunoglobulin expression. Such differences can presumably influence the pathogenesis of disease and hence it clinical presentation but also have implications for the effectiveness of diagnostic serology [21,23,24].

Despite the antigenic similarity of *T. cruzi *and *T. rangeli*, several studies have described species-specific polypeptides allowing specific differentiation of these parasites [25-27]. Our observations account for both the inter- and intra-specific differences observed in IFA, identifying a variety of antigens which are specific not only to species but also to strains and life cycle stages.

Even considering the antigenic differences to blood trypomastigotes, the use of culture-derived trypomastigotes in IFAs in this study significantly reduced the serological cross-reactivity between *T. cruzi *and *T. rangeli *(Figs. 1 and 2). It is clear also, though, that even in trypomastigote forms significant numbers of positive reactive antigens remain (Figs. 3B and 3C).

Regardless of the clinical presentation from which it is obtained, sera from chronic chagasic patients revealed a significantly more intense reaction (p < 0.01) against *T. cruzi *trypomastigotes in IFA when compared with epimastigote forms (Figs. 1 and 2). This difference can be explained by the antibody specificity, since anti-trypomastigote antibodies present on the evaluated sera were produced against blood trypomastigotes during *T. cruzi *infection.

Sera from patients with the indeterminate form of Chagas disease that showed lower titers (1:40 – 1:80) against *T. cruzi *epimastigotes did not recognize *T. cruzi *and *T. rangeli *trypomastigote forms (Fig. 2). Since 76% of the sera from chagasic patients revealed titres equal or in excess of 1:640, but only 1.4% showed titers below 1:160, and since all sera with high titres reacted with both epimastigote and trypomastigote forms of *T. cruzi *and *T. rangeli *we conclude that sera with titers below 1:40 are most likely to be false-positive results.

Thus, considering that Chagas disease diagnosis is primarily based in IFA and/or ELISA assays and that variable result may be obtained depending on the antigens and methods used [7,15,20] the use of culture-derived trypomastigote forms as antigens could reduce misdiagnosis of the disease and the related disability-adjusted life years or DALY's [28,29].

Nevertheless, the use of such infective forms may represent an increased risk to those culturing the organisms as well as an increase in the costs of the diagnostic exams. These drawbacks reinforce the need to generate recombinant antigens or synthetic peptides for specific diagnosis [15].

Previous studies have pointed some species-specific antigens allowing specific detection of *T. cruzi *[30-32] or differentiation of *T. cruzi *and *T. rangeli *[7,25,26] while other antigens seem to be shared by distinct *Trypanosoma *species. Among these studies, Saldaña *et al*. [26,27] have compared total epimastigote extracts from *T. cruzi *and *T. rangeli *by SDS-PAGE and pointed out *T. rangeli *exclusive proteins of approximately 93, 77-73, 63, 54-53 and 48 kDa.

In our present work we have used sera from both acute patients and chronic chagasic patients with distinct clinical presentations (Figs. 3B and 3C), as well as different parasite life-cycle stages (epimastigotes and trypomastigotes), being the first comparative analysis of trypomastigote antigens of both species.

In order to identify possible species-specific antigens present in both *T. cruzi *and *T. rangeli *life-cycle stages, IB assays were initially performed using sera from patients acutely and chronically infected with *T. cruzi*. These experiments addressed the possibility of discriminating stage-specific or clinical presentation-specific antigens. The results indicated at least two proteins present in both *T. cruzi *life cycle stages but were absent in *T. rangeli *(Fig. 3B) and that were strongly recognized by acute and chronic sera. These proteins of approximately 32 and 40 kDa, could represent good targets for the development of specific recombinant antigens, improving the diagnosis of *T. cruzi *in relation to *T. rangeli*. In addition, a 35 kDa protein was exclusively observed on both *T. rangeli *forms extracts and may also represent a new diagnostic marker candidate. However, the amino acid sequence of the exclusive proteins described in the present study as well as their conservation among distinct strains remains to be addressed.

In the present study, the antigenic detection using sera from chronic patients showed the richest protein profile when compared to sera from acute patients reinforcing the results of previous studies [24,30]. However, some proteins bands between 22 and 50 kDa (Fig. 3C) were observed in both parasite species and forms regardless of the sera used. Such proteins may constitute antigens that elicit antibodies production in all phases and/or forms of the disease, which could be good targets to improve differential diagnostic methods or even for vaccine development.

Only around 20% of the *T. cruzi *chronically infected patients develop symptomatic presentations such as cardiac or digestive tract involvement and, despite the efforts for many years, no effective prognostic markers are yet available. Manifestations of Chagas disease range from asymptomatic to subtle abnormalities seen on electrocardiograms which may evolve to advanced congestive heart failure and/or disabling megaesophageal or colonic syndromes. However, the majority (~60%) of infected individuals present no clinical evidence of infection other than positive serology. The reasons leading to such wide variation of clinical presentations are not yet established but are likely related to both host and parasite genetics and to variation in host immunity [33,34]. Using ELISA and western blot assays, Morgan *et al*. [23] reported a difference in the levels of some anti-*T. cruzi *epimastigote antibody isotypes among patients with different clinical presentations, suggesting that the type of antibodies produced by an individual could be related to their clinical presentation.

Serological cross-reactivity between different infectious etiological agents is well reported in the literature. Considering *T. cruzi *infection in humans, cross-reactions may occur in distinct levels with toxoplasmosis, hanseniasis, tuberculosis, auto-immune diseases, leishmaniasis as well as with other trypanosomatid species as formerly reported [15,20,29,30,35].

Recent studies have comparatively tested the use of *T. cruzi *amastigote, epimastigote and trypomastigote forms from two strains as antigens in diagnostic ELISA assays, having observed that trypomastigote forms are as effective as epimastigotes concerning specificity, sensitivity and antigen stability [35,36]. In these studies, the authors discuss the problems involving the use of infective trypomastigote forms versus the possible clinical implications of detecting anti-*T. cruzi *antibodies directed to the bloodstream form, reinforcing the needs for the use of recombinant antigens.

Considering the onus of Chagas disease in Latin and Central America, regarding not only the costs related to treatment and patient care but also the social implications, and further considering the sympatric occurrence with *T. rangeli *in wide geographical areas; the development of a species-specific detection system able to differentiate both parasite species is of utmost relevance [15]. As a result of this study we believe that the use of trypomastigote forms of both species as antigens for IFA will reduce false-positive diagnosis of Chagas disease in areas were *T. rangeli *also occurs. The consequent increment on the costs of trypomastigote-based diagnosis will trade off against a reduction of the costs associated with false-positive results and a direct benefit in terms of human health.

The existing serological cross-reaction between *T. cruzi *and *T. rangeli *due their antigenic similarity is indeed a problem for diagnosis but it may be an interesting point to address protective immunological effects on Chagas disease [8,37,38]. Recently, Basso *et al*. [38] tested the efficacy of *T. rangeli *vaccination in dogs using an emulsion of epimastigote forms from a single strain of the parasite. The challenge with *T. cruzi *blood trypomastigotes on vaccinated animals revealed a reduction of the period and levels of parasitemia, concluding that the protective effect is based on the high antigenic similarity between species and that *T. rangeli *may be a good agent to induce protective immune response against *T. cruzi *in dogs in endemic areas [38]. Despite promising results, this study does not have considered the antigenic variability among distinct strains and forms as well as the possible bias on diagnosis of vaccinated individuals.

Indeed, *T. rangeli *is a good model to address the development of both therapeutic or prophylactic vaccines, however, the antigenic variability among distinct species, strains and forms must be observed while discussing the use antigen-based vaccines [38-40] or even genetic vaccines [41].

Studies on the use of *T. rangeli *as a vaccine candidate for *T. cruzi *infection have reported reduction in parasitemia, clinical symptoms and mortality rates in experimental models [38,39]. However, the use of distinct animal models with specific strains and life-cycle stages of parasite inoculated seem to have influence on the results of both diagnostic and vaccine development assays [15,38].

## Conclusion

In conclusion, we recommend that positive sera for Chagas disease be retested with *T. rangeli *antigens in areas where overlapped distribution of these species occurs. Even considering the drawbacks of associated costs and safety issues on culturing infective forms, our results indicates that the use of both *T. cruzi *and *T. rangeli *trypomastigotes as antigens in IFA or IB assays will help to avoid misdiagnosis of Chagas disease.

Further studies using 2D gel electrophoresis are in progress to obtain the differentially expressed proteins for identification by mass spectrometry. Because of the nature by which they were defined such protein markers will likely prove of interest both diagnostically and biologically.

## Methods

### Serum samples

Approximately 40 serum samples were used for indirect immunofluorescence assays (IFA). Sera from chronic chagasic patients with the cardiac (n = 26) or indeterminate (n = 13) forms were obtained from two distinct cryobanks (Laboratório de Protozoologia, Universidade Federal de Santa Catarina, Florianópolis, SC – Brazil and Centro de Referência para Doenças Infecciosas e Parasitárias, Universidade Federal de Minas Gerais, Belo Horizonte, MG – Brazil). These samples were collected from endemic areas during the 50's and their use was approved by the UFSC Ethics Committee. Four negative serum samples obtained from each institution formed a pool that was used as a single negative control in all assays. All samples had been previously tested as individually negative by IFA using *T. cruzi *epimastigote forms as antigens. Ten additional sera recently obtained from acute chagasic patients (n = 5) [42] and from patients with the digestive form (n = 5) of the chagasic infection were also included in immunoblotting (IB) assays, for which an informed consent approved by the UFSC Ethics Committee was also obtained.

### Parasites

*T. rangeli *Choachi strain and *T. cruzi *Y strain were used for antigen preparation. For IFA, *T. cruzi *CL and *T. rangeli *SC-58 strains were also included. All strains were grown in LIT (Liver Infusion Tryptose) medium supplemented with 15% of fetal calf serum (FCS) at 27.5°C by weekly passages. Epimastigotes forms of both species were obtained in the exponential growth phase in LIT medium. *T. cruzi *culture trypomastigotes were obtained by infection of Vero cells (ATCC-CCL81) in Dulbecco's modified Eagle's Medium – DMEM (SIGMA, St. Louis), pH 7.4 supplemented with 10% FCS. Cells were maintained in 25 cm^2 ^culture flasks (NUNC, Naperville) and infected with Y strain blood trypomastigotes obtained from experimentally infected Swiss mice. *T. rangeli *culture trypomastigotes of the Choachi strain were obtained by *in vitro *differentiation in DMEM as previously described [43] and purified by cation exchange chromatography using a CM-cellulose column [44].

### Indirect Immunofluorescent assay (IFA)

For antigen preparation, *T. cruzi *and *T. rangeli *epimastigotes and trypomastigotes were washed three times in phosphate-buffered saline (PBS) supplemented with glucose 0.1% (PBSG), pH 7.4 and fixed in paraformaldehyde 2% in PBS (v/v) for 2 hours at 4°C. Fixed parasites where then washed twice in PBS pH 7.4, the final concentration was adjusted to 1 × 10^6 ^parasites/ml in PBS supplemented with 1% bovine serum albumin (BSA). The antigens were then distributed (5 μl/well) in 12 well immunofluorescence slides (Knittel, Braunschweig), air dried and stored at -20°C for periods no longer than 2 months.

Each patient sera was individually analyzed using dilutions of 1:40, 1:80, 1:160, 1:320, 1:640 and 1:1,280 in PBS pH 7.4. Positive control sera and negative control serum pool were used at 1:40 dilution. After dilution, 10 μl of each serum was placed on each of the slides and incubated for 30 min at 37°C. After incubation, slides were washed twice in PBS for 10 min and incubated for 30 min with 15 μl/well of a FITC-labelled anti-human IgG conjugate (Biolab, São Paulo) in 0.01% Evan's blue. After a new wash in PBS, slides were then mounted with buffered glycerine under cover slips for microscopic observation with an Olympus Bx40-FL microscope (Olympus, Tokio). Sample titers were determined by the maximum dilution where the fluorescent parasites could be detected. All experiments were performed in duplicate and evaluated by, at least, two independent observers. The obtained results were compared by ANOVA and the means by the Dunn's test, considering significant differences with p < 0.05.

### Protein assays

A total of 1 × 10^8 ^cells of each parasite species, strains and forms were obtained from cultures as described above. Each was washed three times in PBS (10 min/2,000 × *g*) and the pellet stored at -80°C. Also, 10^8 ^uninfected Vero cells cultured as described before were washed three times in PBS (10 min/2,000 × *g*) and the pellet stored at -80°C. The pellets were then ressuspended in 100 μl of lysis buffer (50 mM NaCl, 200 mM Tris-HCl pH 8.0, 1% Nonidet P-40) and 2 μl of a mixture of protease inhibitors (PMSF 44.2 mg/ml, pepstatin 68.6 μg/ml, TPCK 2.0 mg/ml and TLCK 0.5 mg/ml). The samples were then centrifuged at 14.000 × *g *for one hour, at 4°C, and the supernatant containing the protein extracts was stored at -80°C. Protein concentration was determined by the Bradford method, using BSA as a protein standard.

The same amount of proteins extracts from *T. cruzi *and *T. rangeli *epimastigotes and trypomastigotes, as well as from Vero cells, were separated in 12% SDS-PAGE in a 10 cm × 10 cm mini-gel system (Bio-Rad, Richmond) along with a Protein MW Marker (Jena Bioscience, Jena). Briefly, samples of 50 μg of each protein extract were dissolved in an equal volume of sample buffer (125 mM Tris-HCl, pH 6.8, 1.5% 2-mercaptoethanol, 2% SDS, 20% glycerol, 0.1% bromophenol blue) and boiled for 3 min, immediately transferred to ice for 3 min and then resolved at 100 V for approximately 2 h. The protein profiles were visualized by standard Comassie brilliant blue staining and digitally recorded.

The proteins separated by SDS-PAGE were then transferred onto a nitrocellulose membrane (Hybond-ECL, Amersham Biosciences – GE Healthcare) at 25 V overnight in buffer containing 25 mM Tris; 192 mM Glycine and 20% Methanol. After blotting, the membranes were blocked with 5% fat free milk 0.1% PBS-Tween 20 for 1 h, cut as vertical strips of 0.4–0.5 cm wide and individually treated for 90 min at room temperature with each serum sample (acute, indeterminate, cardiac and negative serum pool control) diluted 1:3,000 in 0.1% PBS-Tween 20. The strips were then treated for 90 min with anti-human IgG conjugate labeled with peroxidase (H+L, Promega, Madison) and the reaction developed using the ECL kit (Amersham Biosciences – GE Healthcare) according to the manufacturer's indications.

## Competing interests

The authors declare that they have no competing interests.

## Authors' contributions

All authors have equally contributed on this research and have read and approved the final manuscript.
